# miRNA Expression: I/R Cardiomyocyte and Sevoflurane

**DOI:** 10.3390/biom14121554

**Published:** 2024-12-05

**Authors:** José Luis Guerrero-Orriach, Maria Dolores Carmona-Luque, Guillermo Quesada Muñoz, Maria Jose Rodriguez Capitán

**Affiliations:** 1 Institute of Biomedical Research in Malaga, 29010 Malaga, Spain; guillermo.quesada.m@gmail.com; 2 Department of Anesthesiology, Virgen de la Victoria University Hospital, 29010 Malaga, Spain; la_mariose@hotmail.com; 3 Department of Pharmacology and Pediatrics, School of Medicine, University of Malaga, 29010 Malaga, Spain; 4 Cell Therapy Group, Maimonides Institute of Biomedical Research in Cordoba (IMIBIC), 14004 Cordoba, Spain; mdolorescarmona24@gmail.com

**Keywords:** cardiomyocyte, anesthetic drugs, cardioprotection, sevoflurane, miRNA, ischemia/reperfusion, in vitro model, gene expression

## Abstract

Background: The effects of anesthetic drugs on myocardial cells have been a subject of research for the last 50 years. The clinical benefits of halogenated agents, particularly sevoflurane, have been demonstrated in cardiac surgery patients. These benefits are due to the action of different enzymes and a variety of molecular pathways mediated by the action of small noncoding RNAs (sRNA) such as microRNAs (miRNAs). However, the modulation potential induced by anesthetic drugs on the miRNA expression and their cardioprotective effects is unknown. Objective: To analyze the variation in the expression of a panel of miRNAs induced by halogenated agents to identify their cardioprotective effects. Aims: Variations in the expression of specific miRNAs induce the potential cardioprotective effects of halogenated agents. Methods: An ischemia/reperfusion (I/R) in vitro model of primary human cardiac myocytes (HCMs) was performed. Four study groups were performed: control group (standard culture conditions), I/R group (without hypnotic drugs exposition), I/R-propofol group (I/R-P), and I/R-sevoflurane group (I/R-S). The secretion of p53 and Akt1 cytokines was quantified in the different cell study groups using an Enzyme-Linked ImmunoSorbent Assay, and the differentially expressed miRNAs were identified carrying out a complete genomic sequencing using the Next Generation Sequencing (NGS). Results: HCMs subjected to the I/R procedure and exposed to sevoflurane showed lower secretion levels of p53 factor and higher levels of Akt-1 cytokine compared to HCMs exposed to propofol (p53: I/R-S: 10.43 ± 0.91 ng/mL; I/R-P: 137.92 ± 7.53 ng/mL; *p* > 0.05); (Akt1: I/R-S: 0.62 ± 0.12 ng/mL; I/R-P: 0.23 ± 0.05 ng/mL; *p* > 0.05). The miRNA gene expression analysis (NGS) showed significantly increased expression of the hsa-miR-140-5p and hsa-miR-455-5p, both miRNAs associated with cardiac function; the hsa-miR-98-5p and hsa-miR-193a-5p, both related to apoptosis inhibition; and the hsa-let-7d-5p associated with myocardial protection. This increase was observed in the HCMs group exposed to sevoflurane in comparison to the propofol group. Conclusions: Sevoflurane-induced miRNAs overexpression confers cardioprotection through various mechanisms at the DNA level and the different signaling pathways levels, such as Akt/ERK.

## 1. Introduction

The effects of anesthetic drugs on cardiac cells have been researched in the last 50 years. In the light of the potential benefits of halogenated agents, particularly sevoflurane, their use has spread in patients undergoing cardiac surgery [1,2,3,4,5].

Recently, this potential benefit has been suggested to be mediated by small noncoding ribonucleic acid molecules (smallRNAs), such as the microRNAs (miRNAs) [6,7,8].

Knowledge of miRNAs’ role in heart diseases has advanced in recent years, and their use as biomarkers and therapeutic tools has spread. In contrast, modulation potentially induced by anesthetic drugs and the cardioprotective effects of halogenated agents are totally unknown [9,10].

The only study published to date involving massive miRNA sequencing in cardiac surgery patients is a pilot study of a small sample of patients. In this study, significant differences were observed in miRNA197-3p expression in the postoperative period between patients who received anesthesia and sedation with sevoflurane and those who received propofol. The miRNA197-3p expression increased significantly when propofol was administered as an intravenous anesthetic; however, the miRNA197-3p concentration was significantly higher in the sevoflurane group. Therefore, the role of this miRNA and its modulation by anesthetics are not fully understood. Due to the small sample used, it was impossible to determine variations in the expression of other miRNAs [11].

In this context, we conducted a study to shed light on the potential effects of halogenated agents on the expression of a miRNA’s panel. This study was divided into two series of in vitro experiments including a thorough cellular analysis to assess the potential cardioprotective effects of halogenated agents induced by variations in the miRNA’s expression. Both studies were carried out in primary human cardiac myocytes, so it was not necessary to subject patients to any additional risk to that of the surgical procedure to determine at the cellular level the cardioprotective effects of exposure to hypnotic drugs during the surgical process. The methodology developed was previously validated following a peer review and published [12].

The main objective of the first experimental stage was to determine whether propofol (non-halogenated) or sevoflurane (halogenated) drugs induced miRNAs modulation during myocardial ischemia-reperfusion (I/R). The main objective of the second experimental stage was to identify the role of miRNA197-3p in the promotion and mediation of myocardial damage during I/R procedure, in human cardiomyocytes exposed to both hypnotic drugs. The miRNA197-3p was selected to its potential role as a generator and biomarker of cardiac injury [11].

## 2. Methods

### 2.1. Primary Human Cardiac Myocytes Culture

Primary Human Cardiac Myocytes (HCMs) were purchased from PromoCell^®^ (PromoCell GmbH, Heidelberg, Germany). HCMs were cultured and expanded according to the manufacturer’s instructions. In brief, cryopreserved HCMs in culture passage 2 were thawed in a water bath at 37 °C and transferred to a culture flask containing prewarmed myocyte growth medium (PromoCell GmbH, Heidelberg, Germany) supplemented with the myocyte growth medium kit (PromoCell GmbH, Heidelberg, Germany) and 1% penicillin/streptomycin. Cells were seeded at a density of 15 × 10^3^ cells per cm^2^, and incubated at 37 °C, CO_2_ 5%, and O_2_ 21% in a humidified atmosphere. The dead cells were removed after 24 h, and the culture medium was refreshed at 2–3-day intervals. When the culture reached 80% of confluence, cells were harvested by trypsinization using the Detach Kit (PromoCell GmbH, Heidelberg, Germany) following the manufacturer’s instructions. Cell viability was determined by Trypan Blue solution staining (BioWhittaker Lonza; Verviers, Belgium), and cell counts were performed in a Neubauer chamber. HCMs were reseeded in new flasks and expanded up to passages four and five to conduct the experiments.

### 2.2. Experimental Design

**Figure 1:**
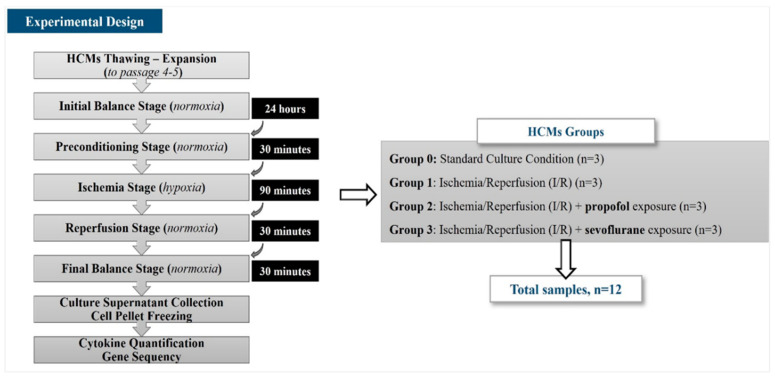
Experimental design.

This in vitro study was designed to analyze the miRNA differential expression in HCMs exposed to halogenated or non-halogenated hypnotic drugs through the Ischemia/Reperfusion (I/R) induction procedure. Four study groups were established: Group 0, as the control group, included HCMs cultured under standard culture condition (*n* = 3); Group 1 included HCMs subjected to the I/R induction procedure and not exposed to hypnotic drugs (*n* = 3); Group 2 included HCMs subjected to the I/R induction procedure and exposed to propofol as a non-halogenated hypnotic drug (*n* = 3); and Group 3 included HCMs subjected to the I/R induction procedure and exposed to sevoflurane as a halogenated hypnotic drug (*n* = 3) (Figure 1).

In this experimental study, the differentially expressed miRNAs were identified by carrying out a complete sequencing of the HCM nucleic acid using the Next Generation Sequencing (NGS) procedure, and the quantification of cytokines secreted by each HCM group was performed with the Enzyme-Linked ImmunoSorbent Assay (ELISA).

### 2.3. Ischemia/Reperfusion (I/R) Procedure and Hypnotic Drugs Exposure

To carry out the exposure of HCMs to hypnotic drugs, and to develop a validated in vitro I/R injury model [13], HCMs were exposed to sevoflurane or propofol as halogenated and non-halogenated hypnotic drugs, respectively, at similar clinical concentrations (280 µM for sevoflurane, similar to 1 minimum alveolar concentration (MAC), and 1 µM for propofol, equivalent to the median effective dose (ED50) applied in surgical procedures). For this purpose, HCMs were cultured in a complete medium enriched with sevoflurane (280 µM) (Sedana Medical AB, Uppsala, Sweden) or propofol (1 µM) (Fresenius Kabi GmbH, Graz, Austria) for 24 h under standard culture conditions as a balance stage. After this, the complete medium was replaced by the same volume of Hank’s Balanced Salt Solution (HBSS) (BioWhittaker Lonza; Verviers, Belgium) supplemented with calcium and magnesium (Capricorn Scientific, Ebsdorfergrund, Germany), cytochalasin B (5 µg/mL) (Sigma Aldrich, St Louis, CO, USA), and sevoflurane or propofol at the same concentration used in the balance stage. HCMs were incubated for 30 min at 37 °C, CO_2_ 5%, and O_2_ 21% in a humidified atmosphere as the preconditioning stage. Once this incubation had been completed, the HBSS solution was replaced with 3 times less volume of the same HBSS enriched solution, and HCMs were cultured under hypoxic conditions (37 °C, CO_2_ 5%, O_2_ 1% in a humidified atmosphere) in a hypoxia chamber (BioSpherix, Parish, NY, USA) for 90 min as the ischemia stage. After that, the hypoxic HBSS was replaced by a normal volume of supplemented HBSS with sevoflurane or propofol, according to the study group, and then, the cells were incubated under standard culture conditions for 30 min. This stage was identified as the reperfusion stage. The HBSS was replaced by a complete culture medium without hypnotic drugs, and the cells were incubated for 30 min under standard culture conditions as the final balance stage.

Finally, HCMs were harvested by trypsinization, washed, and cryopreserved as dry pellets to the gene sequencing assays. The culture supernatants were cryopreserved at −80 °C to the cytokine quantification assay (Figure 1).

### 2.4. Cytokine Quantification

To quantify the cytokine secretion levels of cultured HCMs, the cell culture supernatants were collected once the I/R process was completed, and frozen at −80 °C. The p53 and Akt-1 cytokine levels were quantified. According to the literature, the p53 cytokine increases its expression in cardiac damage [14], and the Akt-1 cytokine secretion enhances cell proliferation, growth, and survival [15]. For this purpose, the Human p53/tumor protein (p53/TP53) ELISA Kit (Cusabio, Houston, TX, USA) and Human Akt1 (RAC-alpha serine/threonine-protein kinase) ELISA Kit (Wuhan Fine Biotech Co, Wuhan, China) were used, respectively. Both protocols were performed according to the manufacturer’s instructions.

### 2.5. RNA Quality for Ultra Sequencing

Once RNA was extracted, RNA was analyzed before sequencing to ensure that it met specific qualitative and quantitative standards. miRNA quality was analyzed by lab-on-a-chip electrophoresis using the Bioanalyzer 2100 kit (Agilent, Santa Clara, CA, USA). The quantification of samples was performed using a Qubit (Life Technologies, Carlsbad, CA, USA) fluorimeter (LifeTechnologies, Carlsbad, CA, USA). Aliquots were stored at −80 °C for later processing.

### 2.6. miRNA-seq and mRNA-seq

For miRNA-seq, the libraries were prepared with NEBNext Small RNA sample preparation (New England BioLabs, Ipswich, MA, USA) following the manufacturer’s instructions. In the case of mRNA-se, Poly-A enriched strand-specific libraries were generated with the TruSeq Stranded mRNA sample preparation (Illumina, San Diego, CA, USA) following the manufacturer’s instructions. The quality and yield of the prepared libraries were assessed using Qubit 2.0 Fluorometer (Life Technologies, Carlsbad, CA, USA), and Agilent 2100 Bioanalyzer (Agilent Technologies, Santa Clara, CA, USA). Library pools were sequenced on a Nextseq550 instrument (Illumina, San Diego, CA, USA) following the manufacturer’s instructions. In the case of miRNA-seq, simple 1 × 75 pb miRNA sequencing was performed for a minimum of 1–10 million reads per sample. For mRNA-seq, 2 × 75 pb sequencing was performed for a minimum of 15–30 million reads per sample.

### 2.7. Analysis Data: Bioinformatics and Statistics

The analysis of mRNA and miRNA gene expression data was performed using the RNA-seq technique. A bioinformatic analysis was performed to determine which mRNAs and miRNAs were differentially expressed between the different groups of samples to be compared. The analysis process involved several stages:

(1) Pre-processing of the reading data to remove from the reading data those nucleotides of low quality, which belong to the adapters of the technique or that come from a source of contamination. For this stage, we used software developed in the Andalusian Bioinformatics Platform (PAB) (University of Malaga, Parque Tecnológico de Andalucía Málaga, Spain) called SeqtTrimNext, v0.9a1, designed with a parallel and distributed operation to speed up the processing of large amounts of data such as those from NGS experiments.
(2) Alignment of the readings to the genomic reference: In this case, the latest version of the human genome (hg38) with an optimized pipeline for the identification of different gene isoforms or the discovery of new genes, TopHat, since it is capable of recognizing exon-intron processing sites. The level of expression was reported in RPKM (Readings Mapped Per Million) format. For the miRNA, the alignment of the reads to the reference was performed, in this case, the latest version of the miRNA (http://www.mirbase.org/) databases with a pipeline optimized for alignment to both the immature and mature forms of the different miRNAs, TopHat. The expression level has been reported in the form of several mapped readings;
(3) In the third stage, the different groups of data were compared to obtain those genes with differential expression. This process involved the pre-normalization of the expression data and the comparison of the data groups. At this stage, we used the tools of the ‘Tuxedo tools’ project, which includes Cufflinks, Cuffmerge, and Cuffdiff tools specially designed to enhance differential expression calculations from RNA-Seq data.

The final results were included in a list of the different differentially expressed genes or miRNAs that meet a series of prerequisites such as stability among the population, minimum level of expression, and statistical significance. The initial determination of miRNAs and mRNAs, through massive sequencing, was conducted by the Ultrasequencing Service and Bioinformatics Service of the Supercomputing and Bioinnovation Center of the University of Malaga (Parque Tecnológico de Andalucía, Málaga, Spain).

For the rest of the statistical analyses, the usual descriptive statistical techniques were used such as Student’s *t*, Fisher’s, or Mann–Whitney *U* tests for the analysis between groups. For comparisons of more than 2 groups, ANOVA or Kruskal–Wallis’s analysis was used. Spearman’s test was applied to study the correlation between quantitative variables. A multiple linear regression analysis was also performed to study the presence or absence of collinearity between variables to determine the validity of bivariate correlations. In any case, Ho’s rejection level was 0.05. All data have been expressed as mean ± standard deviation unless otherwise stated.

## 3. Results

### 3.1. Cytokine Quantification

An analysis was carried out to determine Akt-1 secretion capacity. This factor is directly related to cell proliferation and survival. HCMs were cultured either under standard conditions as the control group, or under I/R conditions and exposed to halogenated (sevoflurane group) and non-halogenated (propofol group) agents to simulate the in vitro I/R procedure in human cardiac myocytes. The last two conditions mimic those of cardiac surgery patients undergoing myocardial conditioning and anesthesia induction. Our in vitro results revealed that Akt-1 factor secretion was lower in the HCMs subjected to I/R processes, regardless of the use or not of anesthetics (I/R group: 0.08 ± 0.02 ng/mL; I/R Propofol Group: 0.23 ± 0.05 ng/mL; and I/R Sevoflurane Group: 0.62 ± 0.12 ng/mL). Thus, Akt-1 factor secretion was significantly higher (*p* ≤ 0.05) in the HCMs cultured under standard conditions (14.74 ± 0.98 ng/mL).

**Figure 2:**
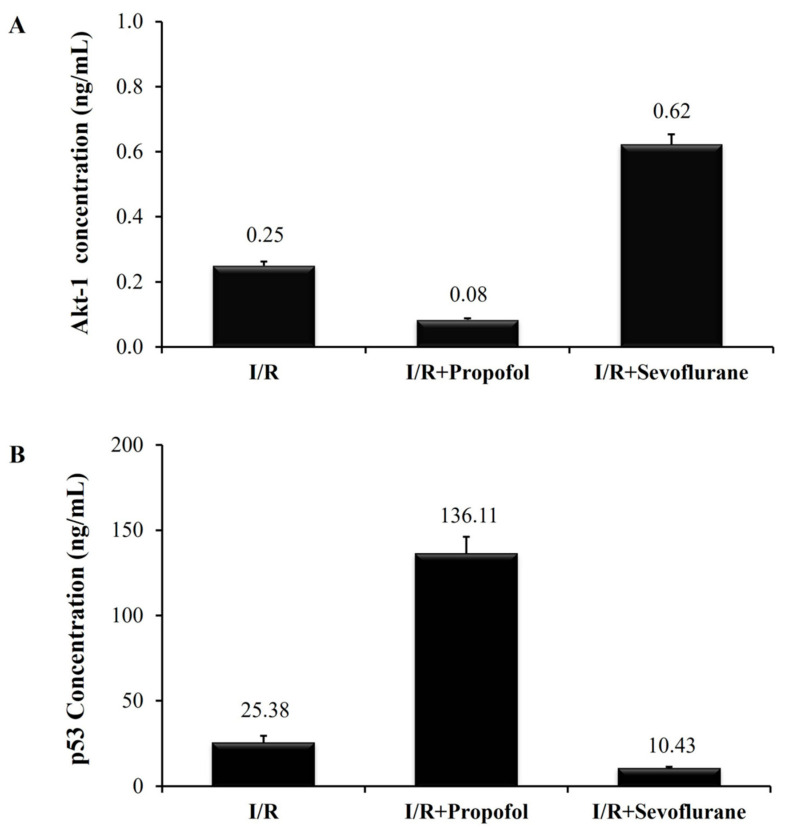
Akt-1 and p53 cytokine quantification. (**A**) Akt-1 supernatant concentration (ng/mL) in the HCM culture. (**B**) p53 supernatant concentration (ng/mL) in the HCM culture. Control group: HCMs cultured under standard culture conditions. I/R group: HCMs culture under hypoxic conditions to simulate the ischemia stage, and under normoxia conditions to simulate the reperfusion stage. I/R-Propofol group: HCMs I/R induced and exposed to propofol as a non-halogenate anesthetic drug. I/R-Sevoflurane group: HCMs I/R induced and exposed to sevoflurane as a halogenate anesthetic drug. *p*-Values were non-significant (*p* > 0.05) (U-Mann–Whitney statistical test).

No significant differences were observed in the Akt-1 secretion capacity of the cells subjected to I/R between the HCMs exposed to sevoflurane and those exposed to propofol (*p* > 0.05) (Figure 2A). These results demonstrate that our in vitro model of I/R induction in HCMs is correctly designed. Hence, our I/R in vitro model generates a reduction of Akt-1 cytokine secretion, which is essential for cell survival and proliferation.

The p53 factor is directly related to myocardial damage. Its secretion was higher in the HCMs subjected to the I/R process (I/R Group: 541.66 ± 3.5 ng/mL; I/R Propofol Group: 137.92 ± 7.53 ng/mL; and I/R Sevoflurane Group: 10.43 ± 0.91 ng/mL), as compared to HCMs cultured under standard conditions. No secretion was detected in the control group (0.00 ± 0.0 ng/mL). Therefore, a statistically significant difference (*p* ≤ 0.05) was observed in p53 secretion levels in the HCMs subjected to I/R not exposed to anesthetics, and HCMs cultured under standard conditions.

This means that p53 secretion capacity was lower in the HCMs subjected to I/R and exposed to sevoflurane, as compared to those exposed to propofol (*p* > 0.05) or unexposed (*p* ≤ 0.05). These results demonstrate that the I/R induction process favors the secretion of cellular damage inducers such as p53. Thus, the HCMs exposed to sevoflurane exhibited lower levels of p53 (Figure 2B).

### 3.2. Analysis of the mRNA and miRNA Gene Expression

There were statistically significant differences between the group that received sevoflurane and the propofol group, especially in six miRNAs and several small RNAs. Table 1.

**Figure 3:**
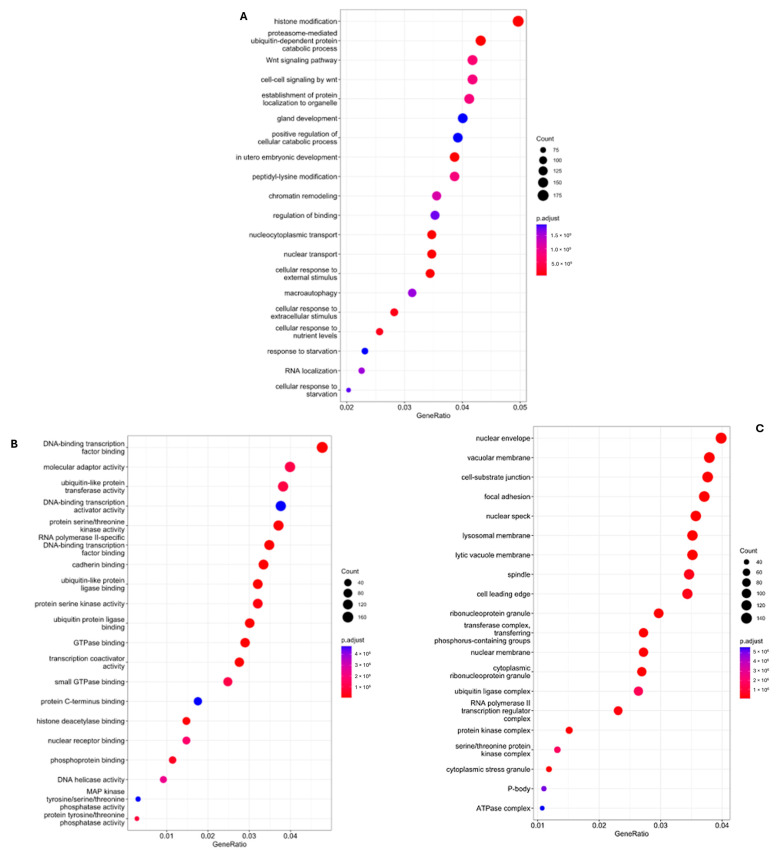
Differences between groups and biological, molecular and cellular components. (**A**) Differences between groups and crucial biological processes involved in miRNA modulation in the sevoflurane group vs. propofol after the ischemia–reperfusion episode; (**B**) Differences between groups and crucial molecular processes involved in miRNA modulation in the sevoflurane group vs. propofol group after the ischemia–reperfusion induction; (**C**) Differences between groups and cellular components involved in miRNA modulation in the sevoflurane group vs. propofol group after the ischemia–reperfusion induction.

Following functional enrichment, gene sequencing revealed how these genes influenced different biological, cellular, and molecular processes. These results have been included in Figure 3A–C.

**Figure 4:**
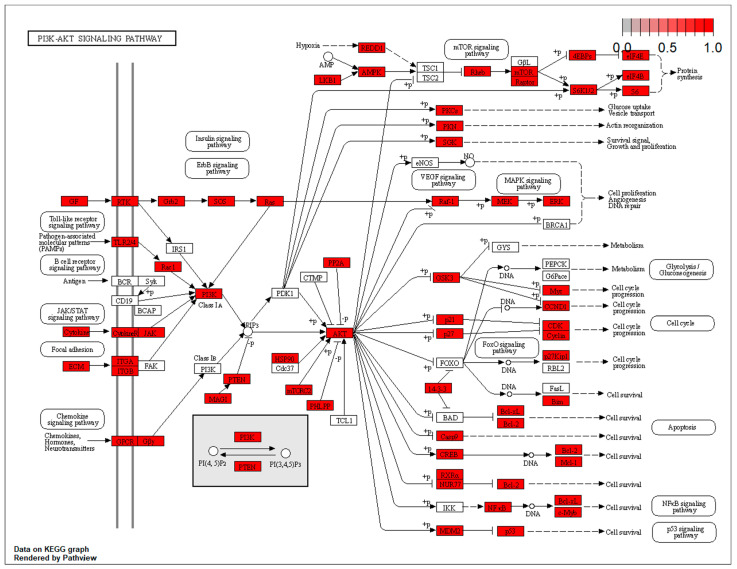
Targets at the level of the AKT/ERK pathways.

**Figure 5:**
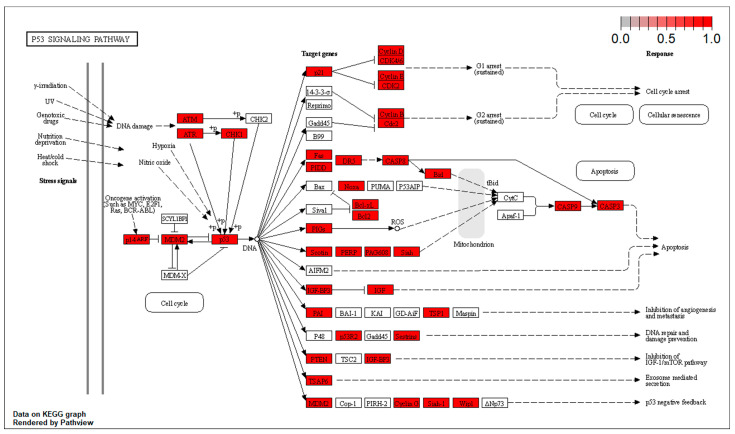
Targets at the level of the p53 pathways.

The targets at the level of the Akt/ERK and the p53 pathways are located at different points of the effector cascade. These results have been included in Figure 4 and Figure 5.

## 4. Discussion

The effect of anesthetics on myocardial cells has been extensively studied in patients, cells, and animal models [16,17,18,19,20]. There is consistent evidence that the cardioprotective effects of sevoflurane, compared to propofol, are mediated by multiple proteins involved in the RISK and SAFE pathways [20,21,22]. No evidence has been provided so far that exposure to anesthetics during I/R procedure induces modifications in smallRNAs, more specifically, in miRNAs.

In our experimental study, we examined differences in a wide variety of smallRNAs.

We found five miRNAs statistically increased, most notably hsa-miRNA193a-5p and hsa-miRNA98-5p.

According to the literature, an increase in the miR193-5p expression is associated with a decrease in myocardial apoptosis. The mechanism by which this upregulation exerts cardioprotective effects has been linked to its effects on the Signal Transducer and Activator of Transcription-3 (STAT3) enzyme [23]. This way induces a decrease in several biomarkers of myocardial damage such as lactate dehydrogenase (LDH), creatine kinase (CK), CK-Myocardial Band (MB), and cardiac Troponin-I (cTnI), and in mediators of the apoptosis in cardiac myocytes such as tumor necrosis factor-α (TNF-α), Interleukin-6 (IL-6), IL-1β, and High Mobility Group Box-1 (HMGB1) proteins. Regarding the effects of miR-193-5p on myocardial contractility, increased levels of STAT3 have been correlated with an increase in the pressure/time index (dp/dt) in myocardial fibers [23,24,25].

In addition, increased miRNA98-5p expression has been reported to suppress apoptosis in cardiac myocytes and reduce the size of myocardial ischemia, and it is associated with an improvement in cardiac function. This effect has been related to the action of this mRNA on Fatty Acid Synthase (FAS) regulation and with the apoptotic signaling via the Caspase-3 pathway. Otherwise, a reduction in miRNA98-5p expression has been associated with an increased size of myocardial ischemia and worsening of cardiac function. Evidence of this beneficial effect has been provided on murine myocardium during the I/R procedure and through the mediation of the DPASK1 protein [26,27].

Other miRNAs involved in cardiac function that showed statistically significant differences in our study were the hsa-miRNA140-5p, hsa-miRNA190-5p, and hsa-miRNA455-5p.

Firstly, the has-miRNA140-5p, identified as a biomarker of acute coronary syndrome, is associated with ischemia induction in the cardiomyocyte. The action of this miRNA is linked to differences in its role at myocardium or plasma levels. At the myocardium level, the miRNA140-5p could inhibit pathological processes in cardiac myocytes induced by drugs such as hypertrophy. This action could be induced by the Mitogen-Activated Protein Kinase (MAPK), which shares its route of action with the Extracellular Signal-Regulated Kinase (ERK) pathway. In contrast, at the plasma level, the miRNA140-5p overexpression has been identified as a biomarker of coronary disease [28].

Secondly, the hsa-miR190-5p has been associated with cardioprotection exerted via the enzymatic modulation of STAT3, which reduces its activity as the expression of hsa-miRNA190-5p increases. The action of this miRNA leads to a reduction of inflammation, myocardial damage, and apoptosis, resulting in the attenuation of cardiac damage during ischemia-reperfusion [29,30].

And finally, the hsa-miRNA455-5p upregulation has been proven to be related to a decrease in ischemia–reperfusion injury, mediated by the SOX2-OT/miR455-3p/TRAF6 target chains. Moreover, the hsa-miRNA455-5p also activates other targets via the MEKK1-MKK4-JNK signaling pathways to protect against ischemia–reperfusion injury [30].

The role of other smallRNAs identified in our study is the subject of ongoing studies due to their modulatory effects being unknown. We could highlight the miRNALet-7d-5p, which mediates a variety of targets related to myocardial protection, High Mobility Group AT-hook-2 (HMGA2) being the most relevant, due to elevated HMGA2 expression possibly leading to the accumulation of DNA damage, inducing Caspase-2 activation, promoting Caspase-3 production, a marker of mitochondria-mediated apoptosis, and inducing apoptosis, although this is dependent on the DNA damage pathway [31,32].

In conclusion, our miRNA differential expression analysis showed that the overexpressed sevoflurane-induced miRNA confers cardioprotection through several mechanisms. This conclusion was supported by the fact that the main targets found in the miRNA’s enrichment process by bioinformatic analysis are directly related to the sites where its effect is exerted on molecular function, with a special role at the DNA level and the level of different signaling pathways, such as Akt (Protein Kinase B)/ERK. It is well known that Akt plays a significant role in DNA damage protection during the ischemia process via the ERK pathway, through regulation of cellular metabolism and senescence. To confirm these findings, we quantified the Akt concentration in the HCM culture supernatant, and the p53 cytokine concentration, related to myocardial damage, both in the sevoflurane and propofol groups. We found that Akt concentration was higher in the sevoflurane group compared to the propofol group, and the p53 concentration was increased in the cardiac myocytes exposed to propofol.

Therefore, we could affirm that the cardiac myocytes exposed to sevoflurane exerted more significant protective effects due to its differential modulatory effects on miRNAs193a-5p, 98-5p, 140-5p, 190-5p, 455-5p, as compared to propofol. It is worth noting that the effect of anesthetics is dual, associated with the triggering of a response at the organ level (the heart, in this case) and plasma level.

Response at the plasma level has been explored in clinical trials in patients undergoing cardiac surgery and in a pilot study that provided results that are inconsistent with ours [11]. This inconsistency may be due to the dual response that these anesthetics induce. However, the protective mechanisms observed in our study act on similar targets to those of previous studies.

The role of the miRNA197-3p, a crucial finding of the study by Guerrero et al., could not be elucidated, as no significant differences were observed between groups in our study. However, a tendency to overexpression was observed in the propofol group. In our opinion, this may be due to the fact that miRNA197-3p might be a mediator of ischemic myocardial injury.

To continue developing new knowledge related to the effect of halogenated drugs on cardioprotection, we have carried out a second experimental study to shed light on the role of miRNA197-3p as a potential cardiac damage biomarker.

Finally, we can conclude that the cardiomyocyte exposure to sevoflurane during myocardial conditioning (I/R procedure) induces overexpression of cardioprotection-related miRNAs and increases the expression of cardioprotective-cytokines and decreases the expression of cytokine related to cell damage.

## Figures and Tables

**Table 1:** RNA sequencing results. Small RNAs and miRNAs that experienced variations before and after ischemia–reperfusion induction in the HCM group exposed to sevoflurane vs. propofol.

Genes	Statistically Significant Difference (*p*-Value)
*ACA20*	0.011
*U65*	0.023
*U79*	0.023
*hsa-let-7d-5p*	0.034
*hsa-miR-127-3p*	0.048
*hsa-miR-140-5p*	0.048
*hsa-miR-193a-5p*	0.048
*hsa-miR-455-5p*	0.048
*hsa-miR-98-5p*	0.048
*hsa-piR-1087*	0.048
*hsa-piR-22236*	0.011
*hsa-piR-25274*	0.034
*hsa-piR-28255*	0.016
*hsa-piR-29204*	0.011
*hsa-piR-32298*	0.034
*hsa-piR-33057*	0.016
*hsa-piR-33115*	0.023
*hsa-piR-33123*	0.023
*hsa-piR-33165*	0.016
*hsa-piR-33185*	0.011
*hsa-piR-3440*	0.016
*tRNA-Gly-CCC-2-2*	0.016
*tRNA-Gly-CCC-chr1-135*	0.016
*tRNA-Pro-AGG-2-4*	0.011
*tRNA-Thr-TGT-3-1*	0.011
*tRNA-Val-CAC-chr1-134*	0.023

## Data Availability

The original contributions presented in this study are included in the article. Further inquiries can be directed to the corresponding author.

## References

[1] Lu Y., Wang L., Liu N., Dong T., Li R. (2016). Sevoflurane preconditioning in on-pump coronary artery bypass grafting: A meta-analysis of randomized controlled trials. J. Anesth..

[2] Li F., Yuan Y. (2015). Meta-analysis of the cardioprotective effect of sevoflurane versus propofol during cardiac surgery. BMC Anesthesiol..

[3] Xu R., Lu R., Jiang H., Li Q.-F., Sun Y., Xu H., Huang Y. (2014). Meta-analysis of the protective effect of sevoflurane on myocardium during cardiac surgery. Eur. Rev. Med. Pharmacol. Sci..

[4] Orriach J.L.G., Belmonte J.J.E., Aliaga M.R., Fernandez A.R., Ponferrada A.R., Navarro M.R., Mañas J.C. (2020). Anesthetic-induced Myocardial Conditioning: Molecular Fundamentals and Scope. Curr. Med. Chem..

[5] Guerrero-Orriach J.L., Carmona-Luque M.D., Raigón-Ponferrada A. (2023). Beneficial Effects of Halogenated Anesthetics in Cardiomyocytes: The Role of Mitochondria. Antioxidants.

[6] Gottlieb R.A., Pourpirali S. (2016). Lost in translation: miRNAs and mRNAs in ischemic preconditioning and ischemia/reperfusion injury. J. Mol. Cell. Cardiol..

[7] Huan T., Rong J., Tanriverdi K., Meng Q., Bhattacharya A., McManus D.D., Joehanes R., Assimes T.L., McPherson R., Samani N.J. (2015). Dissecting the roles of microRNAs in coronary heart disease via integrative genomic analyses. Arter. Thromb. Vasc. Biol..

[8] Sondermeijer B.M., Bakker A., Halliani A., de Ronde M.W.J., Marquart A.A., Tijsen A.J., Mulders T.A., Kok M.G.M., Battjes S., Maiwald S. (2011). Platelets in patients with premature coronary artery disease exhibit upregulation of miRNA340* and miRNA624*. PLoS ONE.

[9] Biglino G., Caputo M., Rajakaruna C., Angelini G., van Rooij E., Emanueli C. (2017). Modulating microRNAs in cardiac surgery patients: Novel therapeutic opportunities?. Pharmacol. Ther..

[10] NNappi F., Singh S.S.A., Jitendra V., Alzamil A., Schoell T. (2023). The Roles of microRNAs in the Cardiovascular System. Int. J. Mol. Sci..

[11] Orriach J.L.G., Belmonte J.J.E., Aliaga M.R., Fernandez A.R., Capitan M.J.R., Muñoz G.Q., Ponferrada A.R., Torres J.A., Santiago-Fernandez C., Gonzalez E.M. (2021). NGS of microRNAs Involved in Cardioprotection Induced by Sevoflurane Compared to Propofol in Myocardial Revascularization Surgery: The ACDHUVV-16 Clinical Trial. Curr. Med. Chem..

[12] Carmona-Luque M.D., Gonzalez-Alvarez L., Orriach J.L.G. (2022). Identification of miRNAs as Biomarkers of Cardiac Protection in Non-Genetically Modified Primary Human Cardiomyocytes Exposed to Halogenated Hypnotics in an In Vitro Model of Transfection and Ischemia/Reperfusion: A New Model in Translational Anesthesia. Life.

[13] Shirai T., Rao V., Weisel R.D., Ikonomidis J.S., Li R.-K., Tumiati L.C., Merante F., Mickle D.A. (1998). Preconditioning human cardiomyocytes and endothelial cells. J. Thorac. Cardiovasc. Surg..

[14] Men H., Cai H., Cheng Q., Zhou W., Wang X., Huang S., Zheng Y., Cai L. (2021). The regulatory roles of p53 in cardiovascular health and disease. Cell. Mol. Life Sci..

[15] Pillai V.B., Sundaresan N.R., Gupta M.P. (2014). Regulation of Akt signalling by sirtuins: Its implication in cardiac hypertrophy and ageing. Circ. Res..

[16] Kunst G., Klein A.A. (2015). Peri-operative anaesthetic myocardial preconditioning and protection—Cellular mechanisms and clinical relevance in cardiac anaesthesia. Anaesthesia.

[17] Su Y., Chen G., Zhang F., Wang L., Feng Z., Gao X. (2021). Isoflurane Alleviates Myocardial Injury Induced by Hypoxia/Reoxygenation by Regulating miR-18a-5p. Cardiovasc. Toxicol..

[18] Mio Y., Uezono S., Kitahata H. (2014). Anesthetic cardioprotection in relation to mitochondria: Basic science. Curr. Pharm. Des..

[19] Zhang J., Wang H., Sun X. (2021). Sevoflurane Postconditioning Reduces Hypoxia/Reoxygenation Injury in Cardiomyocytes via Upregulation of Heat Shock Protein 70. J. Microbiol. Biotechnol..

[20] Orriach J.L.G., Belmonte J.J.E., Fernandez A.R., Aliaga M.R., Navarro M.R., Manas J.C. (2017). Cardioprotection with halogenated gases: How does it occur?. Drug Des. Dev. Ther..

[21] Guerrero Orriach J.L., Ortega G.M., Ramirez Fernandez A., Ramirez Aliaga M., Moreno Cortes M.I., Ariza Villanueva D., Florez Vela A., Torres A.J., Fernandez S.C., Gonzalez M.E. (2017). Cardioprotective efficacy of sevoflurane vs. propofol during induction and/or maintenance in patients undergoing coronary artery revascularization surgery without pump: A randomized trial. Int. J. Cardiol..

[22] Landoni G., Pasin L., Borghi G., Zangrillo A. (2014). Is time to change to halogenated drugs in cardiac surgery, what do we have to do with propofol?. Curr. Pharm. Des..

[23] Pan J., Alexan B., Dennis D., Bettina C., Christoph L.I.M., Tang Y. (2021). microRNA-193-3p attenuates myocardial injury of mice with sepsis via STAT3/HMGB1 axis. J. Transl. Med..

[24] Roy S., Benz F., Cardenas D.V., Vucur M., Gautheron J., Schneider A., Hellerbrand C., Pottier N., Alder J., Tacke F. (2015). miR-30c and miR-193 are a part of the TGF-β-dependent regulatory network controlling extracellular matrix genes in liver fibrosis. J. Dig. Dis..

[25] Wang J.-T., Wang Z.-H. (2018). Role of miR-193a-5p in the proliferation and apoptosis of hepatocellular carcinoma. Eur. Rev. Med. Pharmacol. Sci..

[26] Sun C., Liu H., Guo J., Yu Y., Yang D., He F., Du Z. (2017). MicroRNA-98 negatively regulates myocardial infarction-induced apoptosis by down-regulating Fas and caspase-3. Sci. Rep..

[27] Wronska A. (2023). The Role of microRNA in the Development, Diagnosis, and Treatment of Cardiovascular Disease: Recent Developments. J. Pharmacol. Exp. Ther..

[28] Li X.-D., Yang Y.-J., Wang L.-Y., Qiao S.-B., Lu X.-F., Wu Y.-J., Xu B., Li H.-F., Gu D.-F. (2017). Elevated plasma miRNA-122, -140-3p, -720, -2861, and -3149 during the early period of acute coronary syndrome are derived from peripheral blood mononuclear cells. PLoS ONE.

[29] Li Y., Li Z., Liu J., Liu Y., Miao G. (2021). miR-190-5p Alleviates Myocardial Ischemia-Reperfusion Injury by Targeting PHLPP1. Dis. Markers.

[30] Zhai C.L., Tang G.M., Qian G., Han B.J., Hu H.L., Wang S.J., Yin D., Pan H.H., Zhang S. (2018). miR-190 protects cardiomyocytes from apoptosis induced by H_2_O_2_ through targeting MAPK8 and regulating MAPK8/ERK signal pathway. Int. J. Clin. Exp. Pathol..

[31] Yang Y., Li R., Cao Y., Dai S., Luo S., Guo Q., Wang E. (2020). Plasma MIR-212-3p as a biomarker for acute right heart failure with pulmonary artery hypertension. Ann. Transl. Med..

[32] Wong L.L., Saw E.L., Lim J.Y., Zhou Y., Richards A.M., Wang P. (2019). MicroRNA Let-7d-3p Contributes to Cardiac Protection via Targeting HMGA2. Int. J. Mol. Sci..

